# Seeking allosteric networks in PDZ domains

**DOI:** 10.1093/protein/gzy033

**Published:** 2019-01-23

**Authors:** Candice Gautier, Louise Laursen, Per Jemth, Stefano Gianni

**Affiliations:** 1 Istituto Pasteur—Fondazione Cenci Bolognetti, Dipartimento di Scienze Biochimiche ‘A. Rossi Fanelli’ and Istituto di Biologia e Patologia Molecolari del CNR, Sapienza Università di Roma, Rome, Italy; 2 Department of Medical Biochemistry and Microbiology, Uppsala University, Uppsala, Sweden

**Keywords:** allostery, conformational changes, peptide binding, protein–protein interaction

## Abstract

Ever since Ranganathan and coworkers subjected the covariation of amino acid residues in the postsynaptic density-95/Discs large/Zonula occludens 1 (PDZ) domain family to a statistical correlation analysis, PDZ domains have represented a paradigmatic family to explore single domain protein allostery. Nevertheless, several theoretical and experimental studies in the past two decades have contributed contradicting results with regard to structural localization of the allosteric networks, or even questioned their actual existence in PDZ domains. In this review, we first describe theoretical and experimental approaches that were used to probe the energetic network(s) in PDZ domains. We then compare the proposed networks for two well-studied PDZ domains namely the third PDZ domain from PSD-95 and the second PDZ domain from PTP-BL. Our analysis highlights the contradiction between the different methods and calls for additional work to better understand these allosteric phenomena.

## Introduction

The concept of allosteric interaction was conceived by Umbarger *et al.* in 1956 who discovered a feedback inhibition of l-threonoine deaminase by l-isoleucine (Umbarger and Brown, 1956). The term ‘allostery’ was introduced soon after by Changeux and coworkers and described as an effect, occurring in proteins with several subunits, characterized by an observable conformational change of the quaternary structure upon ligand binding (Monod *et al.*, 1963, 1965; Changeux, 2011). For the last sixty years, experiments and computational studies have broadened this definition to a phenomenon displaying distal changes upon substrate or ligand binding that are not only observable on the quaternary or tertiary structure level but even manifested as a change in dynamics (Cooper and Dryden, 1984; Nussinov and Tsai, 2015). Following this extended definition, allostery has been described in monomeric proteins both between and within protein domains (Cui and Karplus, 2008). Using different approaches, subsets of residues involved in the propagation of energy through the protein (‘allosteric networks’) subsequent to ligand binding have been proposed. The characterization of such allosteric networks in proteins and methods to assess them has recently been thoroughly reviewed by Dokholyan (Dokholyan, 2016). Here, we will focus on the postsynaptic density-95/Discs large/Zonula occludens 1 (PDZ) protein domain family, which emerged as a paradigmatic model system for intradomain allostery because of the work by Ranganathan and coworkers in which pathways of energetically connected amino acid residues were identified by looking at co-evolving residues (Lockless and Ranganathan, 1999; Reynolds *et al.*, 2011; McLaughlin *et al.*, 2012). Intriguingly, theoretical as well as experimental results are conflicting and there is little consensus regarding the nature and role of the proposed networks. In the next sections, we will briefly describe PDZ domains and some of the different methods used to characterize allosteric networks and attempt to highlight the major findings as well as the apparent contradictions.

## PDZ Domains

PDZ domains are protein–protein interaction modules involved in dynamic regulation of signaling pathways and scaffolding (van Ham and Hendriks, 2003; Kim and Sheng, 2004; Ye and Zhang, 2013). They were first observed in synapses of the mammalian nervous system in the early 1990s (Cho *et al.*, 1992) and are now regarded to be one of the most common protein domains in eukaryotes, with 266 identified distinct PDZ domains in the human genome (Luck *et al.*, 2012). They are usually part of multidomain proteins and can form supramodules either of multiple PDZ domains (Ye and Zhang, 2013) or with other domains. PDZ domains have a conserved fold consisting of six beta-strands (β1–β6) and two alpha-helices (α1 and α2). β2 and α2 form a shallow binding pocket in which the ligand peptide arranges as an antiparallel β-strand with β2 (Doyle *et al.*, 1996). The connecting loop between β1 and β2 contains a conserved Gly-Leu-Gly-Phe sequence, referred to as the carboxylate-binding motif because it stabilizes the terminal carboxyl group of the protein ligand via hydrogen bonds from the backbone (Pedersen *et al.*, 2014). This motif is highly conserved and a defining feature when predicting PDZ domains from sequence databases. PDZ domains typically bind C-terminal tails of four to five residues from other proteins with a sequence-based preference. A classification based on peptide ligand specificity has been proposed, but in general the specificities are overlapping (Kang *et al.*, 2003). A significant number of PDZ domains have structured extensions, e.g. the third PDZ domain of PSD-95 (PSD-95 PDZ3; Cooper and Dryden, 1984), the second PDZ domain of NHERF1 (NHERF1 PDZ2; Bhattacharya *et al.*, 2010), the PDZ domain of neuronal nitric oxide synthase (nNOS PDZ; Tochio *et al.*, 2000), the third PDZ domain of ZO-1 (Pan *et al.*, 2011) and the first PDZ domain of harmonin (harmonin PDZ1; Yan *et al.*, 2010). These extensions are proposed to (1) affect protein dynamics-based modulation of target binding affinity, (2) provide binding sites for macromolecular assembly, (3) modulate structural integration of multidomain modules and (4) expand the target ligand-binding pocket (Cooper and Dryden, 1984). NHERF1 PDZ2 and PSD-95 PDZ3 have an α helical extension (α3), which contributes to stability (Wang *et al.*, 2010; Gautier *et al.*, 2017) and ligand binding (Chi *et al.*, 2012), and is suggested to have an allosteric role, because the side-chain dynamics are altered throughout PSD-95 PDZ3 by removing α3 (Petit *et al.*, 2009).

## Methods to Characterize Allostery In PDZ Domains

Over the last two decades, PDZ domains have become a classic model system to study allostery with (e.g. NHERF1 PDZ2) and without (e.g. PSD-95 PDZ3) conformational changes. The allostery is suggested to operate via networks of interactions between amino acid residues. The interaction energy between residues in the network(s) would then define the network. Several computational and experimental approaches have been used to assess the presence and nature of energetic networks in PDZ domains. We start by describing computational approaches such as statistical coupling analysis (SCA), direct coupling analysis (DCA) and rigid-residue scan (RRS), which are often used for predicting allosteric networks computationally. Then, we describe methods such as nuclear magnetic resonance (NMR) relaxation and double mutant cycle (DMC) analysis, which may be used to map allosteric networks experimentally.

### Computational approaches

#### Multiple sequence alignment approaches: statistical coupling analysis

In 1997, Lockless and Ranganathan (1999) proposed to statistically predict allosteric residue networks using multiple sequence alignment (MSA). The method relies on the coevolution principle that postulates that if two residues depend on each other for affording stability, structure or function to the protein and if one of them is mutated, the corresponding interacting residue will also mutate to ensure the preservation of its interaction. Following this assumption, a network of coupled residues could be conserved through evolution within a protein family. Indeed, MSAs can be used to predict tertiary structure of proteins (Rost and Sander, 1993) or refine structures predicted by other methods, showing that spatial proximity of side-chains leads to coevolution (Anishchenko *et al.*, 2017). Lockless *et al.* applied a statistical covariation analysis (SCA) to an alignment of 274 PDZ domain sequences and found that the statistically conserved coupled residues were part of a sparse energetic network mediating peptide ligand binding via an allosteric process. Based on the results, they proposed a general thermodynamic allosteric network for the PDZ family in which three clusters of residues energetically coupled to H372 were identified; the first cluster is in the near environment of H372 (G322, G329, A376 and K380), the second cluster of residues is implicated in ligand recognition (F325 and G322) and the third cluster presents distal residues coupled to H372 through F329 (A347, L353, V362, V386). Development of the SCA approach triggered intense research in elucidating allosteric residues in PDZ domains (Reynolds *et al.*, 2011; McLaughlin *et al.*, 2012) and other proteins (Shulman *et al.*, 2004; Socolich *et al.*, 2005).

The approach has been criticized from various aspects (Fodor and Aldrich, 2004; Anishchenko *et al.*, 2017). For example, binding experiments could not reproduce the original experimental data (Chi *et al.*, 2008) and spatial proximity was shown to explain much of evolutionary covariation in other protein families (Anishchenko *et al.*, 2017). Nevertheless, it is clear that the work by Ranganathan’s lab had the merit of initiating intense multidisciplinary research on this topic.

#### Multiple sequence alignment approaches: direct coupling analysis

DCA is a second approach applying MSA to map residues in close contact, either within a structure, between subunits or in a protein–protein interaction. The method requires two steps: (1) identification of correlated residues obtained from the covariance analysis by MSA using maximum-entropy modeling and (2) separation of direct versus indirect interactions from the initial correlation data. Separation of direct versus indirect correlation (Morcos *et al.*, 2011) is an advantage of the method as compared to simple correlation analysis (Anishchenko *et al.*, 2017). DCA thus identifies residue pairs proximal in space in the folded protein (Weigt *et al.*, 2009), which enhances the accuracy of the contact map used for structural prediction solely from sequences. Hence, the power of DCA is prediction of direct structurally coupled residues. In contrast, SCA predicts both distal and proximal coupled residues (Lockless and Ranganathan, 1999). Even though the two approaches use MSA as input data they do not necessarily predict the same close-contact residue pairs, because SCA uses clustering analysis whereas DCA uses maximum-entropy modeling, as illustrated by comparing DCA and SCA predictions for serine proteases (Morcos *et al.*, 2011). Therefore, it is important to be aware that DCA and SCA may extract different coevolved residue pairs. Baker and coworkers tested the confidence of MSA approaches to predict coevolved spatial or direct residue pair (Anishchenko *et al.*, 2017). They used 3883 proteins, which have at least 1000 distinct sequences in the database for the MSA analysis. Based on the study, they suggest that evolutionary coupled residue pairs are spatially proximal, either within or between protein domains. They also tested the idea that spatially separated residues are coupled if they are part of an allosteric network. However, they see little evidence for long-range allosteric coupling for residue pairs as proposed by SCA and the existence of conserved allosterically coupled residue pairs remains under debate (Süel *et al.*, 2003; Gianni *et al.*, 2011; Hultqvist *et al.*, 2013; Anishchenko *et al.*, 2017).

#### Deep coupling scan

MSA approaches are often used to deduce the internal pattern of energetically or structurally coupled residues in a protein family. However, such theoretical approaches have the limitation that it is difficult and costly to experimentally verify them. Recently, Ranganathan and coworkers reported a case-study for the PDZ family, that experimentally corroborated the pattern of coevolved amino acids as predicted by SCA (Salinas and Ranganathan, 2018). Deep coupling scan (DCS) (Olson *et al.*, 2014), an extended deep mutagenesis approach, was applied at nine residues from the helix α2 in five PDZ homologs. In the study, they created a library of all single and double mutants and probed the effect on binding for each variant by a bacterial two-hybrid assay. Every mutant was quantified by relative enrichment in cell growth by deep sequencing before and after selection. In the assay, PDZ ligand binding induces transcription of a reporter gene that enables growth in presence of an antibiotic. DCS was only applied to a small part of the PDZ domain (α2), due to the exponential expansion of mutants with sequence length. Because of the high correlation between experiment from DCS and theoretical data from DCA or SCA the authors suggested that DCS is a benchmark for future test and validation of theoretical models for protein function. The authors concluded that MSA is a valid method to detect intradomain allosteric pathways without the need to perform experiments, if the mathematical principles of SCA (energetic) and DCA (contact) are unified.

#### Anisotropic thermal diffusion

Ota and Agard developed a molecular dynamics method, anisotropic thermal diffusion (ATD) to probe the hotspot residues implicated in the intramolecular energetic network of PSD-95 PDZ3. ATD is an energy-based computational method in which a target residue is locally heated and the heat propagation is monitored. It was applied to PSD-95 PDZ3 using His-372 as the target heated residue and the results reveal a pathway from His-372 to Ile-341, Ala-347 and Leu-353 via Ile-327 and Phe-325. This publication proposes Ile-327 as a third hotspot, the first two being His-372 and Phe-325 already identified by Lockless et al. (Ota and Agard, 2005).

The method proved to be a cheap, powerful and complementary approach to experimental mapping of the intramolecular allosteric signaling pathway (Burendahl and Nilsson, 2012). The approach was later applied to five PDZ domains with similar backbone fold, but different sequence and ligand-binding specificity (Ho and Agard, 2010). A simple relationship between the computed energetic couplings and conformational flexibility upon ligand binding in α helices was observed. PTP-BL PDZ2 and PSD-95 PDZ3 with and without conformational change upon ligand binding were used in the case-study. PSD-95 PDZ3 with rigid α helices reports coupling between the α helix and the body of PDZ, whereas no coupling was observed for PTP-BL PDZ2 with flexible and ligand responsive α helices. On the basis of these findings, a model was proposed suggesting that α helices in PDZ domains are intrinsically dynamic, but the dynamics is reduced if residues interact at key tertiary contacts.

#### Rigid-residue scan

RRS is a molecular dynamics simulation approach used to systematically identify residues important for intramolecular communication (Kalescky *et al.*, 2015). RRS probes the role of each residue in overall dynamics of the protein by performing separate simulations for every amino acid applied with rigid body constrain. Every residue is separately perturbed by the ‘rigid body constrain’ meaning that the residue is made rigid while keeping the rest of the protein flexible to reveal its contribution to overall protein dynamics. A heat map with cross correlation matrix for unperturbed versus perturbed residue is used in the analysis to allow identification of two groups of residues with different functions: (1) ‘switches’, which are required to initiate the binding effect from protein allostery and (2) ‘wire residues’, which propagate energy or information from the binding site to distal locations within the protein. PSD-95 PDZ3 was used as a case-study for rigid-residue scan to deduce the intramolecular network. Eight of the nine identified residues in PSD-95 PDZ3 have been reported previously with other methods as important for the intramolecular allosteric network: G329, I336, I338, A347, H372, A390, Y397, F400. Recently, the method was extended with entropy analysis to deduce the relationship between each residue and overall protein dynamics to explain the allosteric mechanism (Kalescky *et al.*, 2016). Entropy analysis was performed for every RRS simulation in bound and unbound state to deduce the entropy contribution from each residue. The residue with the rigid body constrain loses all internal degrees of freedom, which increases the flexibility in other parts of the protein resulting in an overall increase of the entropy of the protein following Le Châtelier’s principle. This extended method was applied to PTP-BL PDZ2 as a case-study and 11 key residues showed significant protein entropic response to rigid body perturbation (Kalescky *et al.*, 2016). Seven of the eleven residues have been identified in a previous NMR study (Fuentes *et al.*, 2004) to be important for the intramolecular allosteric network within PTP-BL PDZ2: D15, L18, T28, R31, V40, L78 and T81. The authors therefore suggested that RRS is a systematic approach to deduce the contribution from internal degrees of freedom in every residue to protein dynamics and allostery.

#### Interaction correlation method

Molecular dynamics interaction correlation analysis is a method that shows conceptual similarities to MSA-based studies but relies on different information. While the MSA approach proposed by Lockless and Ranganathan (1999) is based on the alignment of proteins with homologous sequences, the interaction correlation method compares different conformers of one protein as obtained by MD (Kong and Karplus, 2009). In particular, a residue correlation matrix is built from the interaction energy correlations between residue–residue pairs in an ensemble of protein conformers generated by nanosecond MD simulations. While this method is computationally more expensive, it has been proposed to depict the energetic pathways of a single protein domain with higher accuracy than MSA used for SCA, which relies mainly on sequence alignments. Kong and Karplus (2009) applied the interaction correlation method to PTP-BL PDZ2 and identified two contiguous interaction pathways. One pathway starts at the binding site on strand β2 and goes along the N-terminal side of helix α1 and the adjacent C-terminus of loop β1–β2. The other pathway starts at the binding site and extends perpendicularly across strands β2, β3, β4, β6 and β1 towards the opposite side of PTP-BL PDZ2.

### Experimental approaches

#### NMR relaxation

Among the experimental approaches, NMR has the advantage that it can measure both structure and dynamics. Thus, NMR relaxation experiments have been used to assess dynamics of proteins and observe entropically driven allosteric processes (Popovych *et al.*, 2006). ^2^H spin relaxation studies the dynamics of the side-chain methyl groups of proteins whereas ^15^N spin relaxation experiments provide information on the backbone dynamics. Relaxation data fitted with the Lipari–Szabo model give rise to two main parameters useful to evaluate fluctuations in the picosecond–nanosecond timescale (Lipari and Szabo, 1982). The order parameter S^2^ represents the amplitude of the bond’s movement and varies from 0 to 1, 1 corresponding to a complete rigidity of the bond; and the τ_e_ parameter indicates the correlation time for the bond motion. Other models can be used to fit the relaxation data and when relaxation rates observed are high the data should be fitted using models including *R*_ex_ which represents the motion on a microsecond–millisecond timescale (Cooper and Dryden, 1984). Andrew Lee’s group used NMR relaxation together with binding and site directed-mutagenesis experiments to reveal key residues involved in propagation of allosteric signals in PTP-BL PDZ2 and PSD-95 PDZ3 (Fuentes *et al.*, 2004, 2006; Petit *et al.*, 2009). They found that long-range dynamic effects can be observed directly by NMR and have successfully identified two distal clusters: the first one situated in the region of β4 and β5 and the second one in helix α1 (Fuentes *et al.*, 2004). NMR relaxation parameters have also been used as restraints in molecular dynamics simulations revealing networks of residues presenting changes in dynamics and structures upon peptide binding, e.g. in PTP-BL PDZ2 (Dhulesia *et al.*, 2008). How well do the NMR relaxation data correlate with the residues proposed by SCA? Ten residues are statistically coupled to H372 according to SCA (Lockless and Ranganathan, 1999). Fuentes et al. (2006) tested the one corresponding residue pair in PTP-BL PDZ2 (H71-I20), but did not find any thermodynamic coupling. According to Fuentes *et al.*, the absence of the coupling between these residues in PTP-BL PDZ2 is a reminder of the different potentials of the methods: (1) SCA reports trends for a whole protein family, which may not apply to all proteins in the same family (2) NMR relaxation reports thermodynamic coupling between two residues based on mutagenesis studies in a specific protein, which can affect the comparison if the same mutations are not used to probe the effect.

#### Double mutant cycles

DMC analysis uses thermodynamic cycles to study folding and binding processes of proteins and has the power to quantitatively determine the energetic connectivity between any two residues. Carter et al. (1984); Horovitz and Fersht (1992) initially developed the DMC methodology for probing intramolecular interactions, but the method was subsequently successfully used to study intermolecular interactions between a protein and its ligand (Schreiber and Fersht, 1996). In a DMC analysis, the binding free energy of the wild type protein is compared with those of two single mutants at positions A and B and of the corresponding double mutant (both A and B being mutated). If the effects on thermodynamics of the single mutations do not sum up to the effect of the double mutant, then we can define a coupling free energy (ΔΔΔGC), which represents the free energy of the interaction and is defined as the difference of the double mutant binding free energy with the two single mutants binding free energies. Thus, if ΔΔΔGC ≠ 0, the two mutated residues are energetically coupled. On the other hand if the effects on thermodynamics of the single mutations sum up to the effect of the double mutant, then the coupling free energy equals zero and the residues are not energetically coupled. The DMC approach has been applied by our groups on three different PDZ domains to map energetic networks and thus providing a comparison of homologous proteins: PTP-BL PDZ2, SAP97 PDZ2 and PSD-95 PDZ3 (Chi *et al.*, 2008; Gianni *et al.*, 2011; Hultqvist *et al.*, 2013). Our data did not support an evolutionarily conserved network of energetically coupled residues. Instead, we observed that several energetic pathways are sampled within one single domain and distinct pathways are activated by specific protein ligands, highlighting the complexity of PDZ allostery (Hultqvist *et al.*, 2013).

## Comparing the Energetic Networks in PDZ Domains

Prompted by the work of Lockless and Ranganathan (1999), intense research was triggered in elucidating the network of residues involved in the allostery of PDZ domains and gave rise to many publications in which different results were obtained. However, as highlighted above, an outlook on the different studies appears rather complex, both because of the different methodologies applied and because of the different protein systems taken into account. Therefore, a systematic approach to analyse our current knowledge on the presence and structural localization of allosteric networks in PDZ domains would demand a critical comparison on the same protein systems.

**Fig. 1:**
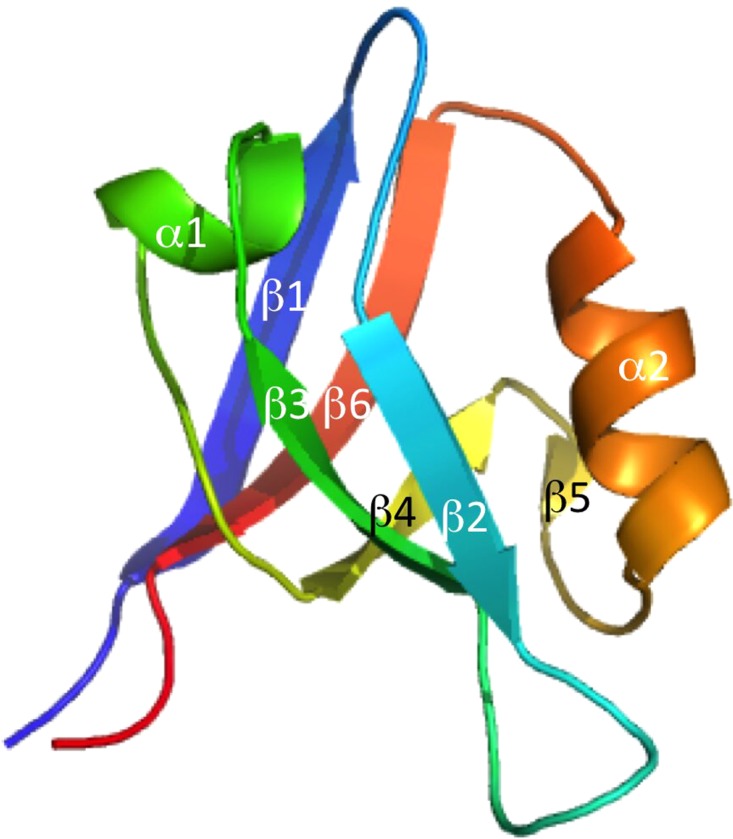
Three-dimensional structure of a canonical PDZ domain, PTP-BL PDZ2. The secondary structure elements are labeled according to the standard nomenclature used in PDZ domains.

**Fig. 2:**
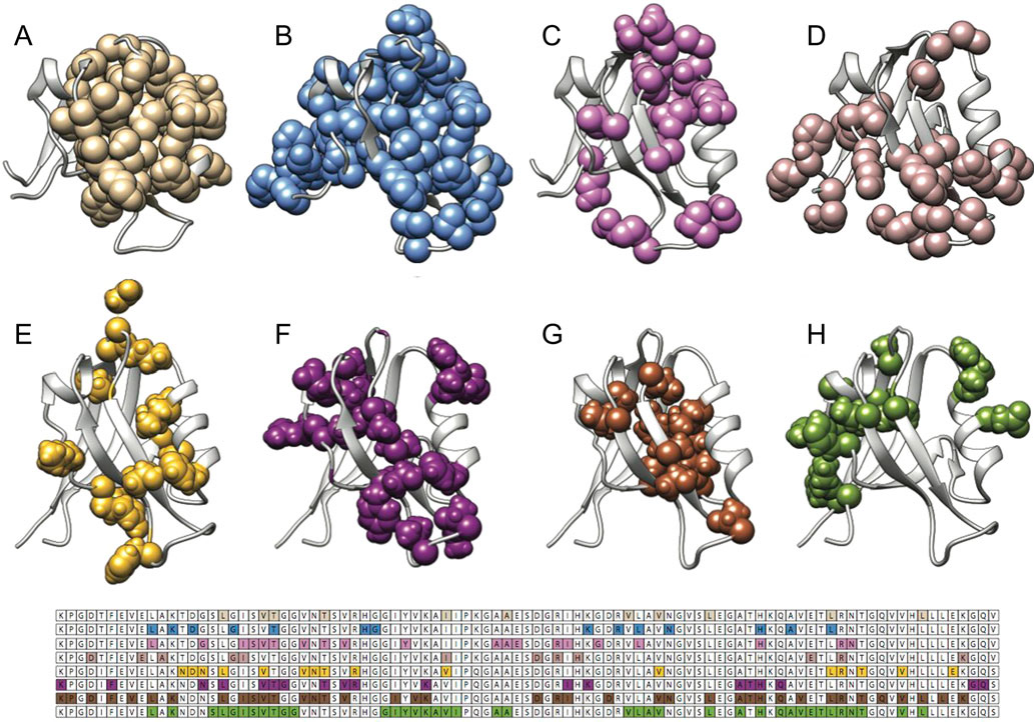
Allosteric networks in PTP-BL PDZ2 determined by different approaches. Allosteric networks mapped in mouse and human PTP-BL PDZ2 (pdb 1GM1 and pdb 3LNX) with different methods: (**A**) Thermodynamic double mutant cycle (Gianni *et al.*, 2011) (**B**) NMR, relaxation (Fuentes *et al.*, 2006) (**C**) NMR, relaxation, in combination with Molecular dynamics simulations (Dhulesia *et al.*, 2008) (**D**) NMR (^15^N HSQC spectra upon titration) (van den Berk *et al.*, 2007) (**E**) Rigid-residue scan (RRS) (Kalescky *et al.*, 2016) (**F**) Machine learning models (Kalescky *et al.*, 2016) (**G**) Protein structure network and elastic network model (PSN-ENM) (Raimondi *et al.*, 2013) (**H**) Perturbation response scanning (PRS) (Gerek and Ozkan, 2011).

Two well-studied PDZ domains, which have been subjected to several studies on their allosteric networks, are PTP-BL PDZ2 and PSD-95 PDZ3. Figs 1 and 2 report a graphical representation of the different residues that have been identified to play a role in the allosteric network of PTP-BL PDZ2 and PSD-95 PDZ3, respectively. Remarkably, inspection of Figs 1 and 2 reveal that a comparison between the allosteric networks pinpointed using different methods returns different results, with the allosteric networks being dependent on the choice of method used to detect it. Thus, whilst certain hotspot positions appear to be robust, a clear set of conserved distal residues dynamically changing upon ligand binding remains to be identified. Furthermore, when merging all the networks previously mentioned together, more than 50% of the amino acids in both PTP-BL PDZ2 and PSD-95 PDZ3 are suggested to be allosteric. This notion suggests that, in agreement with some recent observations (Law *et al.*, 2017; Hayatshahi *et al.*, 2018), in the case of PDZ domains, allostery may involve a prominent fraction of the structural architecture of the domain.

**Fig. 3:**
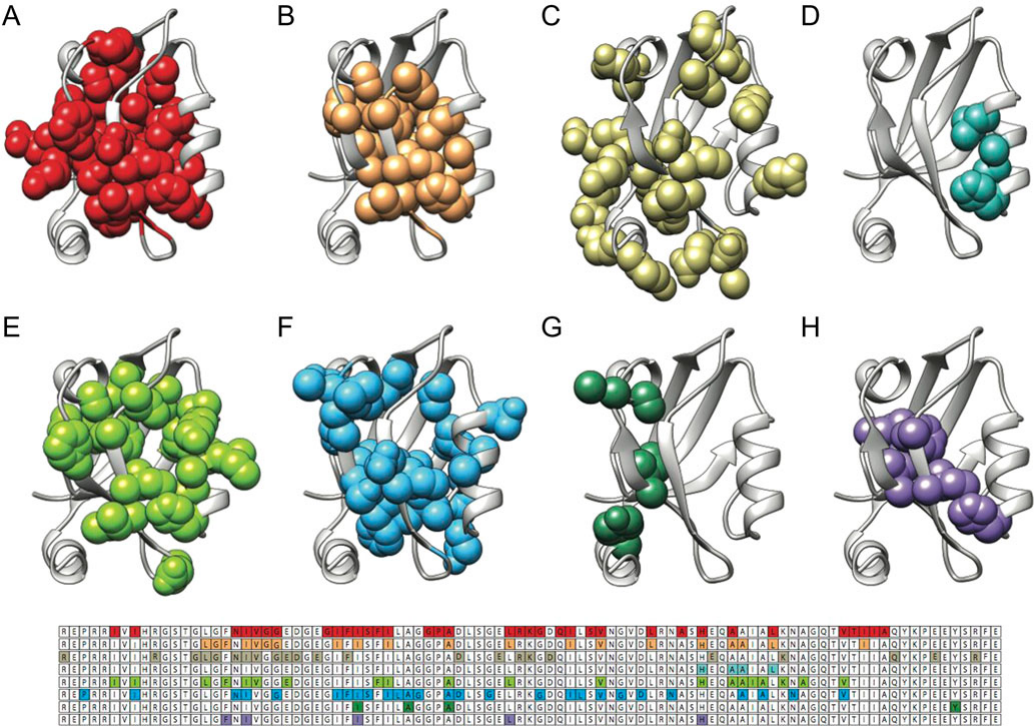
Allosteric networks in PSD-95 PDZ3 determined by different approaches. Allosteric networks mapped in human PSD-95 PDZ3(pdb 1be9) with different methods: (**A**) Perturbation response scanning (PRS; Gerek and Ozkan, 2011) (**B**) Statistical coupling analysis (SCA) (McLaughlin *et al.*, 2012) (**C**) Molecular dynamics simulation (Kumawat and Chakrabarty, 2017) (**D**) Deep coupling scan (DCS) (Salinas and Ranganathan, 2018) (**E**) Thermodynamic double mutant cycle (TDMC) (Gianni *et al.*, 2011) (**F**) Conservation mutation correlation analysis (CMCA) (Du *et al.*, 2010) (**G**) Rigid-residue scan (RRS; Kalescky *et al.*, 2015) (**H**) Monte Carlo path (MCPath; Kaya *et al.*, 2013).

The puzzling comparative result reported in Figs 2 and 3 highlights the inherent complexity of describing the allosteric networks in a protein domain. In fact, whilst several experimental and computational methods converge in stressing a role of intradomain allostery within the PDZ moiety, the structural details of such effects are far from understood. Given that the same PDZ domain may display different networks depending on the specific ligand (Hultqvist *et al.*, 2013), it is not clear at this stage whether the discrepancies observed in Figs 2 and 3 are related to the inherent limitations of the different methods rather than to a specific complexity of the PDZ domain family. It is evident that more work is needed to understand this scientific problem, possibly involving the collaborative efforts of different research group applying complementary experimental and computational methods on the same domain.

## Conclusions

On a more general level, what can we learn from the extensive body of work on energetic networks in PDZ domains? It is clear that the choice of method influences the result, which is likely a result of the relatively weak energetic connectivity and complexity of amino acid residue interactions involved in the networks. In comparison to for example protein folding, which is relatively robust to perturbation due to the funnelled energy landscape, allostery mediated by small conformational changes, or changes in dynamics, appears less robust and more prone to remodeling. It is clear, therefore, that the understanding of such allosteric pathways demands the synergy between different experimental and computational methods to be fully understood.
